# Chromosome number variation and phylogenetic divergence of East Asian *Cirsium* sect. *Onotrophe* subsect. *Nipponocirsium* (Compositae), with a new species from Taiwan

**DOI:** 10.1186/s40529-025-00454-2

**Published:** 2025-02-14

**Authors:** Chih-Yi Chang, Pei-Chun Liao, Hsy-Yu Tzeng, Junko Kusumi, Zhi-Hui Su, Yen-Hsueh Tseng

**Affiliations:** 1 Taiwan Forestry Research Institute, No. 53, Nanhai Rd., Zhongzheng Dist., Taipei, 10066 Taiwan; 2 School of Life Science, National Taiwan Normal University, No. 88, Sec. 4, Ting-Chow Rd., Wenshan Dist. 116, Taipei, Taiwan; 3 Department of Forestry, National Chung-Hsing University, No. 145, Hsing-Ta Rd., Taichung, 402 Taiwan; 4 Department of Environmental Changes, Faculty of Social and Cultural Studies, Kyushu University, Fukuoka, 819-0395 Japan; 5 JT Biohistory Research Hall, Takatsuki, Osaka 569-1125 Japan; 6 Department of Biological Sciences, Graduate School of Science, Osaka University, Osaka, 560-0043 Japan

**Keywords:** Descending dysploidy, Phylotranscriptomics, Polyploidization, Pollen morphology, Taxonomy

## Abstract

**Background:**

This study explored chromosome number variation, phylogenetic divergence, and mechanisms underlying speciation in East Asian thistle *Cirsium* Mill. sect. *Onotrophe* (Cass.) DC. subsect. *Nipponocirsium* Kitam. (Compositae). The study focused on the newly identified species from Taiwan: *Cirsium pengii* Y.H. Tseng, P.C. Liao & Chih Y. Chang. Utilizing phylotranscriptomic data to reconstruct evolutionary relationships between the Taiwanese and Japanese taxa of *Cirsium* subsect. *Nipponocirsium* as well as their divergence times and chromosomal characteristics. Additionally, the chromosome number, morphology, and pollen morphology of the unknown *Cirsium* taxon are compared with other known subsect. *Nipponocirsium* taxa from Taiwan.

**Results:**

Phylotranscriptomic analysis reveals a division within subsect. *Nipponocirsium* into Japanese and Taiwanese clades. In the Taiwanese clade, *C. pengii* is basal, while *C. tatakaense* remains monophyletic with other Taiwanese species despite higher genetic diversity. The prevalent chromosome number in this subsection is tetraploid (2*n* = 4*x* = 68), common in Japanese taxa, while Taiwanese members have 2*n* = 4*x* = 64. Notably, *C. pengii* has a diploid number (2*n* = 32), indicating descending dysploidy followed by polyploidization in Taiwan. This polyploidization, driven by glaciations, likely shaped the evolution of *Nipponocirsium*. Divergence time estimates suggest the separation of Japanese and Taiwanese clades around 0.74 million years ago (Myr) during glacial periods. *Cirsium pengii* diverged around 0.47 Myr, while tetraploid species *C. kawakamii* and *C. tatakaense* diverged around 0.35 Myr. These species likely evolved in separate refugia, with distinct species boundaries confirmed through species delimitation analysis, karyotype, morphology, and pollen morphology comparisons.

**Conclusions:**

These findings enhance our understanding of chromosomal evolution and speciation within subsect. *Nipponocirsium* and underscore the importance of integrating transcriptomic data in phylogenetic studies. This study provides a comprehensive framework for further investigations into the genetic diversity and adaptive mechanisms of this ecologically vital group.

**Supplementary Information:**

The online version contains supplementary material available at 10.1186/s40529-025-00454-2.

## Background

Polyploidy, a common and recurrent phenomenon (Van de Peer et al. 2017), is widely observed in angiosperms (Soltis et al. 2009; Schmidt-Lebuhn et al. 2010; Cannon et al. 2015; One Thousand Plant Transcriptomes Initiative 2019; Bureš et al. 2023). Polyploidy events in the ancestral lineage of seed plants have profoundly shaped their evolutionary trajectory; incorporating these events into phylogenetic analyses elucidates patterns of speciation and adaptation, offering deep insights into angiosperm evolution (Soltis et al. 2009; Jiao et al. 2011; Amborella Genome Project 2013; Van de Peer et al. 2021). Polyploidy, which drives reproductive isolation, plays a role in nonadaptive radiations (Gorelick and Olson 2013). In addition, polyploidy can promote species diversification and evolutionary innovation (Soltis et al. 2009; Cannon et al. 2015; Van de Peer et al. 2017). However, the resulting whole-genome duplications create complex genetic histories that must be accurately revealed to enable understanding of the evolutionary relationships among species.

Changes in chromosome number and structure are significant processes that often reflect speciation events, as they can establish reproductive barriers between populations (Schubert and Vu 2016; Winterfeld et al. 2020). Bureš et al. (2023) stated that descending dysploidy and polyploidization are common in the subtribe Carduinae (Compositae), and it exhibit strong phylogenetic signals. When individuals with different chromosome numbers interbreed, their offspring often have reduced fertility or viability; this results in a reproductive barrier that can prevent gene flow, eventually leading to the emergence of new species (Walsh 1982; Bozdag and Ono 2022). We previously observed a distinct chromosomal count (2*n* = 32) in the Taiwanese thistle *Cirsium taiwanense* Y.H.Tseng & Chih Y.Chang; this count was lower than that in its close relatives *C. arisanense* Kitam. and *C. hosokawae* Kitam. (2*n* = 34). This suggests the occurrence of descending dysploidy, which promotes speciation, in *Cirsium* within Taiwan (Chang et al. 2021).

*Cirsium* Mill. (Compositae) comprises approximately 250 extant species, including perennial, biennial, and annual spiny herbs; these herbs are characterized by capitula containing only disc florets, involucres arranged in 5–20 series, setiferous receptacles, five synantherous stamens with sharply short-tailed anther bases, and plumose pappi (Keil 2006; Funk et al. 2009). According to the literature, 46 species of *Cirsium* are present in China (Shih and Greuter 2011), 64 in Japan (Kadota 1995), and 9 in Taiwan (Peng 1998). We recently found that the population of native *Cirsium* species in Taiwan has expanded to include 11 species, 3 varieties, and 1 form, totaling 14 taxa, along with one naturalized plant (Chang and Tseng 2019, 2020; Chang et al. 2019, 2021). Notably, the highest species diversity was observed in regions with mid to high elevations, seven species and one form, all endemic to Taiwan (Peng 1998; Chang et al. 2019, 2021).

The infrageneric classification of Eastern Asian *Cirsium* (Kitamura 1937; Shih 1984; Kadota 1995, 2007), Taiwanese taxa belonged to three sections: Sect. *Pseudoeriolepis* (Nakai) Kitam. and sect. *Spanioptilon* (Less.) Shih had only one species respectively, and sect. *Onotrophe* (Cass.) DC. had the remaining taxa. Sect. *Onotrophe* is further divided into four subsections: subsect. *Arenicola* Kitam., subsect. *Australicirsium* Kitam., subsect. *Nipponocirsium* Kitam., and subsect. *Sinocirsium* Kitam. Among these subsections, subsect. *Nipponocirsium* is characterized by the absence of rosette leaves or their withering at anthesis as well as the presence of cauline leaves that are typically pinnatipartite or pinnatisect, U-shaped spaces between the pinnae, and nodding mature capitula (Kadota 1995; Peng 1998; Chang et al. 2019; Kadota and Miura 2021), which is differ from other subsection, there are 6 species in Japan (Kadota 1995), and 2 species in Taiwan (Chang et al. 2019).

According to Ackerfield et al. (2020a), recent phylogenetic analyses of entire *Cirsium* indicate that, although *Cirsium* as currently circumscribed is polyphyletic, the Asian and American members form a well-supported monophyletic group. Taiwanese taxa include *C. lineare* (sect. *Spanioptilon*), which is phylogenetically located within a relatively basal clade (Funk et al. 2009; Ackerfield et al. 2020a, b). Although other Taiwanese taxa were not included in these analyses, species closely related to the Taiwanese taxa, such as *C. nipponicum* (Maxim.) Makino (subsect. *Nipponocirsium*), *C. japonicum* DC. (subsect. *Sinocirsium*), and *C. henryi* (Franchet) Diels (probablely belongs to sect. *Spanioptilon*), are positioned in progressively more derived clades (Ackerfield et al. 2020a).

The chromosome number 2*n* = 34, which is strongly predominant among species of *Cirsium* (Hsu 1970; Peng and Hsu 1978; Masukawa et al. 1990; Bureš et al. 2004; Özcan et al. 2008, 2011), is considered the ancestral diploid number (Bureš et al. 2004; Mota et al. 2016; Ackerfield et al. 2020a). A small number of species exhibit different chromosome numbers, such as 2*n* = 30, 32, 64, and 102 (Bureš et al. 2010; Chang et al. 2019, 2021; Ackerfield et al. 2020a). In Eastern Asia, the basic chromosome number for *Cirsium* is found not only *x* = 17 (Frankton and Moore 1961; Hsu 1970; Ownbey et al. 1975; Peng and Hsu 1978; Polat et al. 2018) but also *x* = 16 and 14 (Ackerfield et al. 2020a).

Within the genus, tetraploidy (2*n* = 4*x* = 68) is relatively uncommon and has derived independently through polyploidization events in only a few lineages, such as *C. nipponicum* (subsect. *Nipponocirsium*) (Ackerfield et al. 2020a). In Japan, almost all species of the genus possess a chromosome number of 2*n* = 4*x* = 68 (Masukawa et al. 1990; Kadota 1995; Kadota and Miura 2014, 2021). By contrast, all species in Taiwan have a chromosome number of 2*n* = 4*x* = 64, hypothesizing the occurrence of descending dysploidy (Chang et al. 2019). In plants, polyploidization, which results from genome doubling, often promotes growth (Bamford and Winkler 1941; Kulkarni and Borse 2010; Van de Peer et al. 2021). This results in relatively larger plant bodies in spescies of subsect. *Nipponocirsium* than in other diploid *Cirsium* spescies. The species of *Cirsium* in Taiwan that underwent descending dysploidy during evolution indicate an evolutionary pathway distinct from their Japanese relatives.

**Fig. 1:**
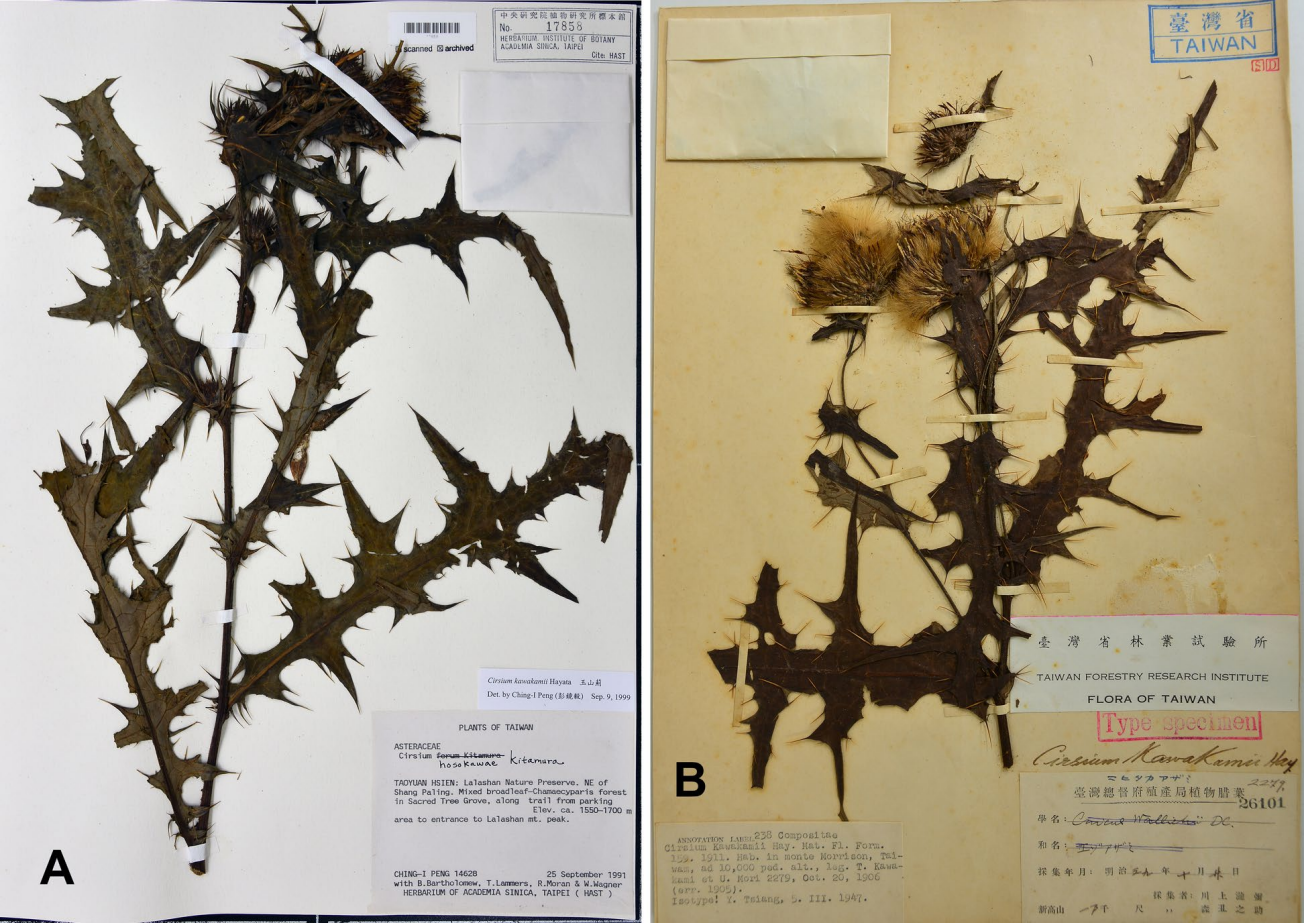
Comparison of specimen: (**A**) the unknown *Cirsium* (*Peng, Chin-I 14628*, HAST 17858) and (**B**) the isotype of *C. kawakamii* Hayata (*Kawakami T. & Mori U. 2279*, TAIF 26101)

During our recent field and herbarium investigations in the Mt. Lalashan area in New Taipei City, Taiwan, we discovered an unknown taxon. This taxon was subsequently identified to be *Cirsium kawakamii* Hayata (*Peng, Ching-I 14628*, Herbarium of the Agricultural Science Department [HAST 17858]; Fig. 1A). Compared with *C. kawakamii* (Fig. 1B), this new taxon had smaller leaves, shorter leaf lobes, and a smaller plant size. However, the nodding mature capitula, cauline leaves (lack of rosette leaves), and U-shaped area between the leaf lobes suggest that the new taxon belongs to subsect. *Nipponocirsium*, along with *C. kawakamii* and *C. tatakaense* Y.H.Tseng & Chih Y.Chang (Kitamura 1937; Kadota 1995; Chang et al. 2019). However, the unknown taxon has small leaves and plant bodies, which suggests its diploid nature. To gain deeper insights into the evolution of subsect. *Nipponocirsium*, we investigated its karyotype and phylogenetic relationships with closely related species. In the present study, we elucidated the phylogeny and karyotype of subsect. *Nipponocirsium* across Japan and Taiwan, examined variations in chromosome numbers, and determined the taxonomic status of the unknown taxon.

Transcriptomic data were used to reconstruct phylogenetic relationships and estimate divergence times. Subsequently, species delimitation analyses were performed for the specimens. Transcriptome analysis is a cost-effective method for obtaining RNA coding sequences (Wang et al. 2009b). These sequences not only encode specific active proteins but also provide insights into gene sequences undergoing evolutionary selection, thereby revealing phylogenetic relationships (Wang et al. 2009b; Martin and Wang 2011; Cheon et al. 2020). Cheon et al. (2020) demonstrated that phylogenetic relationships determined using transcriptomic data are as reliable as those reconstructed using genomic data. To determine the taxonomic status of the unknown taxon, we compared its karyotype, morphology, and pollen morphology with those of other known subsect. *Nipponocirsium* taxa from Taiwan.

## Methods

### Taxon sampling, RNA extraction, and RNA sequencing

We sampled 13 individuals representing seven *Cirsium* species, including all 3 species of subsect. *Nipponocirsium* from Taiwan (2 to 3 individuals per species) and three species from Japan (single individuals per species). Two individuals of *C. lineare* (Thunb.) Sch.Bip from Taiwan were included as the outgroup. To improve the calibration points (CPs) for estimating the divergence time, transcriptomic data of sequence read archive (SRA) of the following four species were downloaded from the National Center for Biotechnology Information (NCBI) website (Supp. Table 1): *Silybum marianum* L. (SRR12539244) and *Cynara cardunculus* L. (SRR16295441) from Cardueae, *Gerbera delavayi* Franch. (SRR5480948) from Mutisieae, and *Nastanthus ventosus* (Meyen) Miers. (SRR12034794) from Calyceraceae.

Fresh leaves were collected and immediately preserved in RNAlater Solution (Tissue RNA Protecting Reagent; catalog number: TRP010.500; Bioman Scientific, New Taipei City, Taiwan) at – 20 ℃. Total RNA was extracted using a modified cetyltrimethylammonium bromide (CTAB) method (Doyle and Doyle 1987; Logemann et al. 1987; Gambino et al. 2008; Schenk et al. 2023). Polyvinylpyrrolidone (PVPP) and a high-concentration sodium chloride solution were added to the CTAB buffer to remove polyphenols and polysaccharides, respectively (Logemann et al. 1987; Schenk et al. 2023). Double-stranded complementary DNA was synthesized from the extracted RNA and sequenced on the Illumina NovaSeq 6000 platform; this process generated read lengths of up to 2 × 150 bp. Illumina raw reads were trimmed using Trimmomatic v0.39 (Bolger et al. 2014). Raw sequence reads have been submitted to the SRA of NCBI under the BioProject ID PRJNA1158676.

### De novo assembly and orthologous gene identification

Each sample was subjected to de novo assembly by using the method described by Freedman and Weeks (2021). Ribosomal RNA (rRNA) sequences were removed using Bowtie 2 v2.4.2 (Langmead and Salzberg 2012) by aligning them against the Silva online rRNA database (Quast et al. 2013). Overrepresented sequences were eliminated using the script “RemoveFastqcOverrepSequenceReads.py” (Freedman and Weeks 2021). Transcriptome sequences were assembled using Trinity v2.12.0 (Grabherr et al. 2011). Duplicate genes were removed using CD-HIT-EST v4.8.1 (Li and Godzik 2006; Fu et al. 2012); the sequence identity threshold was set to 0.85. The quality of transcriptome assemblies was assessed using BUSCO v5.3.2 (Manni et al. 2021) against the Embryophyta odb9 database. Coding sequences were then predicted using TransDecoder v5.5.0 (Brian and Papanicolaou 2018).

Orthologous genes (OGs) were identified, and gene copy numbers were determined using OrthoFinder v2.5.4 (Emms and Kelly 2019). Sequences in each gene cluster were aligned using macse v2.06 (Ranwez et al. 2011). Subsequently, ambiguous sites were trimmed using trimAl v1.4.1 (Capella-Gutiérrez et al. 2009) with the following parameters: *-gt 0.2 -seqoverlap 80 -resoverlap 0.8*.

### Phylogeny reconstruction

Phylogenetic relationships were reconstructed using both multispecies coalescent and concatenated methods. Single-copy OGs shared by at least 85% of the samples were selected for phylogenetic and Splitstree analyses, and all samples were included in the Bayesian analysis. For the multispecies coalescent method, individual gene trees were estimated using IQ-Tree v2.3.3 (Nguyen et al. 2015) with extended model selection followed by tree inference (*-m MFP*), and 1,000 bootstrap replicates (*-B 1000*). These gene trees were then utilized to infer the species tree by using Astral v5.7.7 (Mirarab and Warnow 2015) with default parameters. The resulting species tree was edited and visualized using FigTree v1.4.3 (Rambaut et al. 2014); the tree was rooted at *N. ventosus* for all samples and at *C. lineare* for the *Cirsium* taxa subset.

To meet the computational demands of BEAST v2.6.3 (Heled and Drummond 2010; Bouckaert et al. 2019), we selected the longest 50 genes for analysis. A BEAST run was conducted using the StarBeast3 module (Douglas et al. 2022) in BEAUti v2.6.3 (Bouckaert et al. 2019). For each gene, we used a strict-clock model, a ploidy value of 2.0, and a site model determined using the Akaike information criterion in jModelTest v2.1.10 (Guindon and Gascuel 2003; Darriba et al. 2012). We used the same parameter settings described by Bagley (2016). A total of 100 million generations and trees were logged every 1000 generations by using the Yule model and default priors in BEAUti. Convergence and effective sample size values (> 200) were verified using Tracer v1.7.1 (Rambaut et al. 2018). Topology was visualized using DensiTree v2.2.7 (Bouckaert 2010). The maximum clade credibility tree was summarized using TreeAnnotator v2.6.3 (Rambaut and Drummond 2014), with a 10% burn-in of logged trees. The final species tree was edited and visualized using FigTree v1.4.3 (Rambaut et al. 2014).

For the concatenated method, aligned gene sequences were combined into a supermatrix by using FasParser v2.13.0 (Sun 2017). Neighbor-net networks were inferred using Splitstree v4.18.1 (Huson and Bryant 2006) with default settings, and *C. lineare* was omitted to prevent long branch attraction.

### Species delimitation

Species delimitation was exclusively performed within *Cirsium* taxa to minimize interference from outgroups. Individual trees were used to validate species boundaries by using discovery methods (Carstens et al. 2013). A rooted Astral species tree was analyzed using SODA v1.0.2 (Rabiee and Mirarab 2021), with a threshold of 0.001 applied. The single-rate Poisson tree processes (PTP) model was used through a Web server (https://mptp.h-its.org/), with a *p*-value of 0.001 (Kapli et al. 2017). The Bayesian version of PTP (bPTP; https://species.h-its.org/ptp/) (Zhang et al. 2013) was run with default settings. The Generalized Mixed Yule Coalescent (GMYC) approach required an ultrametric, bifurcating tree free of zero-length branches (Fujisawa and Barraclough 2013; Kapli et al. 2017); a Bayesian species tree was used to fulfil these requirements. We resorted to a Web server (https://species.h-its.org/gmyc/) to analyze the data by using both single- and multi- threshold approaches with default settings.

### Divergence time estimation

The settings for the BEAST run were similar to those used in the previously described phylogenetic reconstruction methods. To enhance the calibration point (CP) for estimating divergence time, taxa additional to those in the *Cirsium* genus were included (Supp. Table 1), and two CPs were set. CP1 corresponded to the origin of Compositae (83.5 Myr) (Mandel et al. 2019; Ackerfield et al. 2020a). By contrast, CP2 was established at 14 Myr, which was derived from achene fossils identified as *Cirsium* (Mai 2001) and positioned between the nodes of *Cynara* and *Silybum* (Ackerfield et al. 2020a). The major glacial events and their durations during the Quaternary Period were determined from a study conducted by Gradstein et al. (2012) and are indicated on the time scale.

### Morphological comparison

The morphology of all known Taiwanese taxa of subsect. *Nipponocirsium* taxa, including *C. kawakamii*, *C. tatakaense*, and the unknown taxon. The comparisons were performed using both fresh and herbarium specimens. Herbarium acronyms follow Index Herbariorum (Thiers 2024, continuously updated). Voucher specimens collected for the current study were deposited in TAIF, TCF, TNM, and HAST. Herbarium sheets or images from the following herbaria were examined: HAST, KYO, PPI, TAI, TAIF, TCF, TI, and TNM. The Taiwanese taxa were compared with similar Japanese taxa, including *C. suffultum* (Maxim.) Matsum. and *C. nipponicum* (Maxim.) Makino var. *incomptum* (Maxim.) Kitam., and *C. kujuense* Kadota (Kadota 1995, 2006, 2008).

### Pollen morphology comparison

Pollen morphology of the unknown taxon was compared with those of *C. kawakamii* and *C. tatakaense* (Supp. Table 1). For each voucher, 3 to 6 pollen grains were measured. Pollen grains from fresh material were directly mounted on a stub without pretreatment and sputter-coated with gold (Quorum SC7620) for observation under a scanning electron microscope (Hitachi S-3400N). The shape, size, and exine ornamentation were analyzed following methods outlined by Erdtman (1952) and Halbritter et al. (2018).

### Morphological data analysis

Quantitative morphological and palynological traits are expressed as mean ± standard deviation values. Differences between taxa were analyzed using a one-way analysis of variance, followed by Tukey’s honestly significant difference multiple-range test (Zar 1984). All analyses were performed using PASW Statistics v.18 (Sarma and Vardhan 2018).

### Chromosome number analysis

Living plants were cultivated in greenhouses at the Department of Forestry, National Chung Hsing University, Taiwan. Root tip samples were collected on sunny mornings and pretreated with 2 mM 8-hydroxyquinoline at a temperature < 4   for 8 h. Subsequently, the samples were fixed in Carnoy’s solution (absolute ethanol/acetic acid ratio: 3:1 [v/v]) at− 7 °C for at least 24 h.

Chromosome preparations were conducted using the squash method and the flame-drying method. For the squash method, chromosomes were prepared following Özcan et al. (2011) and Yüksel et al. (2013), with minor modifications. Samples were stained with 2% acetic orcein in 1N HCl at room temperature for 48 h. After staining, the samples were squashed and observed under an optical microscope (Accu-Scope 3025) equipped with a CCD camera (ProgRes C14 plus).

For the flame-drying method, chromosome preparations followed the protocols of Kurata et al. (1981) and Lee et al. (2020), incorporating minor modifications to the enzyme maceration and flame-drying technique. Root tips were macerated in a solution of 2% cellulose (Onozuka R-10, Yakult Honsha, Tokyo) and 2% pectinase (Sigma Chemical Co.) in 10 mM citrate buffer (pH 4.5) at 37 °C for 30 min. The macerated material was squashed on slides using a fixative (methanol:acetic acid = 3:1, v/v). Slides were flame-dried, air-dried, and stained with 4',6-diamidino-2-phenylindole (DAPI) in an antifade solution (VectaShield Mounting Medium, Vector Laboratories, CA, USA). Images were captured digitally using a CCD camera mounted on an epifluorescence microscope (Axioskop 2, Carl Zeiss AG, Germany). Voucher materials are presented in Supp. Table 3. From each voucher, 8–10 metaphase plates were analyzed.

## Results

### Bioinformatics analysis of transcriptome sequencing, de novo assembly, and OG identification

The raw sequencing data obtained from the 13 samples totaled 683,798,752 reads, with an average of 52,599,904 reads per sample and an average GC content of 47%. For 4 samples acquired from the NCBI, the raw data totaled 106,623,206 reads, with an average of 26,655,802 reads per sample and an average GC content of 45%. In total, the 17 aforementioned samples yielded 843,021,862 reads, with an average of 49,589,521 reads per sample and an average GC content of 46%. After trimming, rRNA removal, and overrepresented sequence elimination were completed, approximately 322.8 million high-quality reads remained, with an average of 19.0 million reads per sample and an average GC content of 45%. The average proportion of complete BUSCOs across the 17 samples was 72.86%, and the proportion of duplicate genes was 33.01%. After the removal of duplicate genes, the average proportion of complete BUSCOs slightly decreased to 72.60%, and the proportion of duplicate genes decreased to 12.26% (Supp. Table 1).

A total of 44,539 OGs were identified from the 17 samples. From these OGs, we selected 3,037 single-copy OGs shared by 85% of the samples. After aligning these sequences and trimming ambiguous sites, we retained 2569 OGs with a cumulative length of 2,527,911 bp and an average length of 984 bp per OG. Furthermore, 44,751 OGs were identified from the 13 *Cirsium* samples. From these, we selected 4054 single-copy OGs shared by 85% of the samples. After alignment and trimming, we retained 3321 OGs, totaling 3,152,925 bp with an average length of 949 bp per OG. These retained genes were used to infer individual gene trees, which were then coalesced to reconstruct phylogenetic relationships.

### Phylotranscriptomic insights

**Fig. 2:**
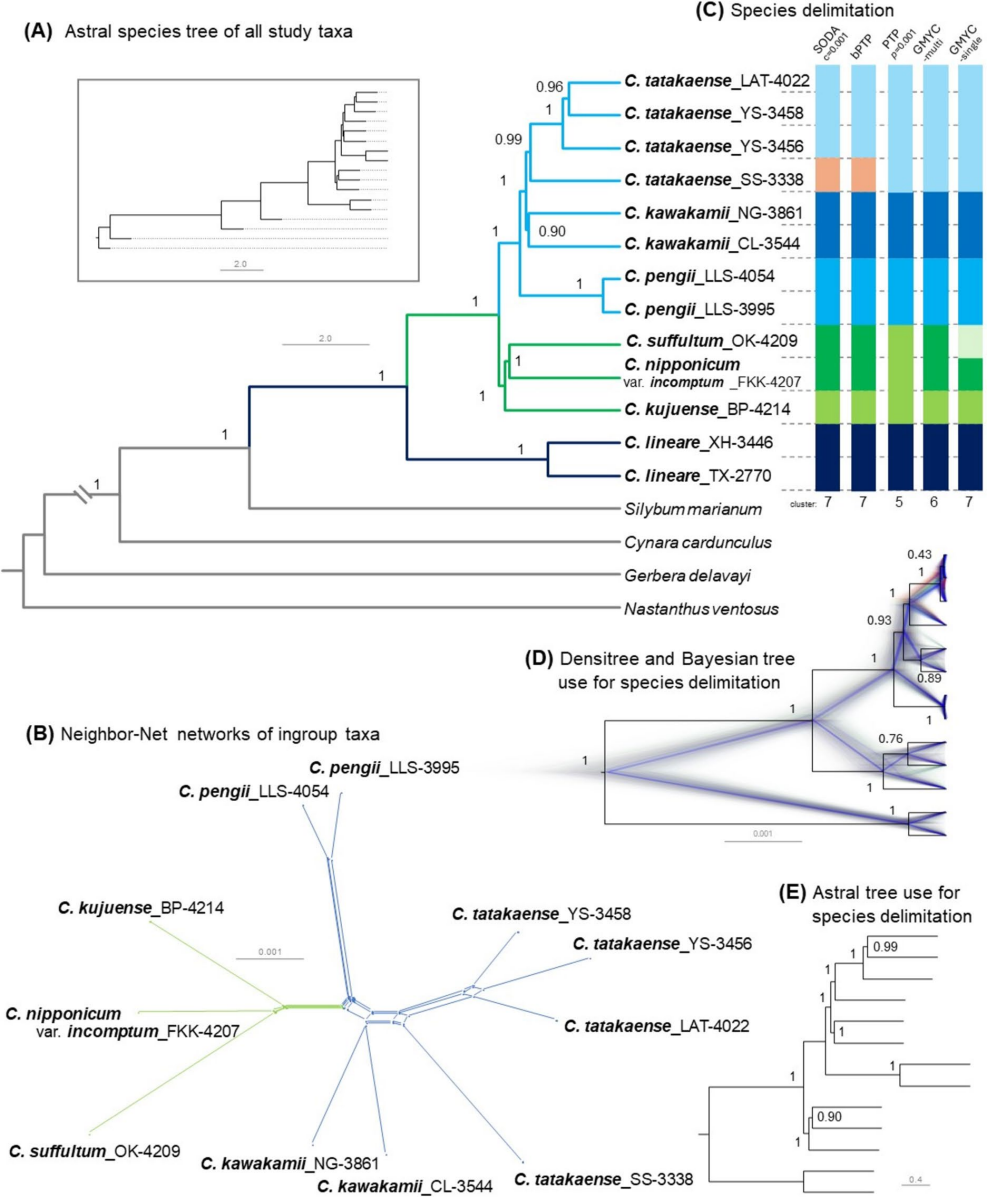
Phylotranscriptomic analysis of *Cirsium* Mill. subsect. *Nipponocirsium* Kitam. **A:** Astral species tree of all study taxa, using coalesced single-copy sequences from 2,569 OGs; within the box is the complete topology. **B:** Neighbor-Net networks of ingroup taxa based on concatenated single-copy sequences from 3,321 OGs. **C:** Species delimitation analysis. The GMYC analyses are based on Bayesian tree (**D**); The SODA and PTP analyses are based on Astral species tree (**E**); different colors represent distinct clustering results. **D:** Densitree and the consensus tree of *Cirsium* taxa constructed from the posterior distribution of trees from a Bayesian analysis. Note that the densitree reveals conflicting signals that cannot be captured in a single tree. **A:** Grey clades represent outgroups; deep blue clades indicate outgroups within *Cirsium*; **A**, **B:** green clades represent taxa native to Japan; blue represents taxa native to Taiwan. **A, D, E:**The numerical values of node annotations represent posterior probabilities (PP)

**Fig. 3:**
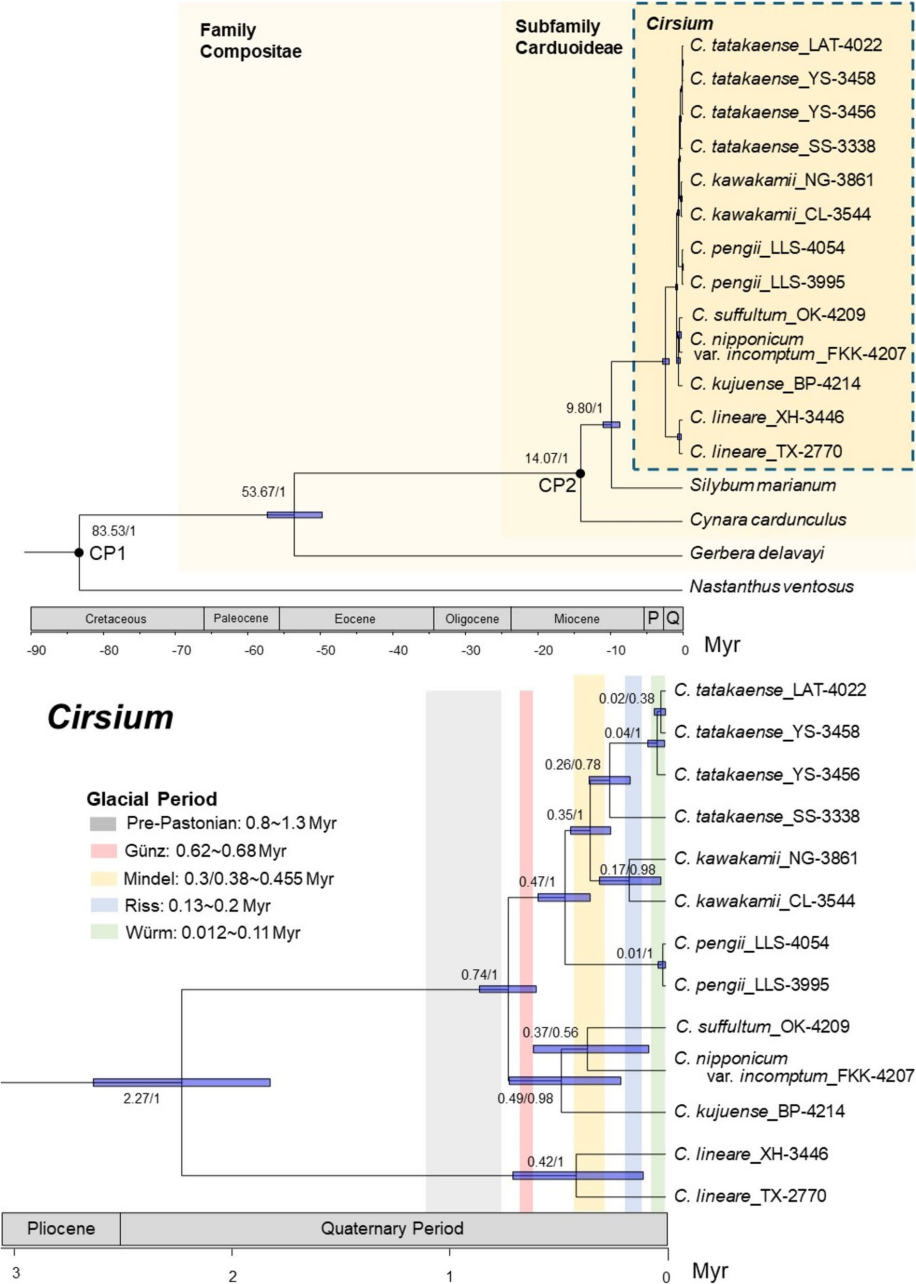
Time-calibrated phylogeny of *Cirsium* Mill. subsect. *Nipponocirsium* Kitam. within Compositae. Node number indicate median age/posterior probabilities (PP). Blue bars on nodes indicate the 95% confidence intervals (CI). Black dots on nodes represent the calibration points (CP) utilized in the dating analysis. In the scale axis, “P” and “Q” correspond to Pliocene and Quaternary, respectively. Different color blocks represent each glacial period

All phylogenetic analyses results exhibit a similar topologies and robust node support (Fig. 2). Except for Bayesian analysis (Fig. 2D, Fig. 3), which exhibits lower posterior probability (PP) for the intraspecific clade of *C. tatakaense* (PP = 0.43 [Fig. 2] and 0.38 [Fig. 3]). As indicated in Fig. 2D, conflicting signals observed within the intraspecific clade of *C. tatakaense* could not be fully resolved in a single tree. This limitation may be attributable to the extensive computational demands of BEAST; incorporating additional genes may increase the difficulty of achieving convergence. Nevertheless, the topology of the consensus tree was consistent with other results (PP = 0.96 [Fig. 2A] and 0.99 [Fig. 2E]), indicating the reliability of the overall findings. In summary, our findings reveal clear divergence and well-established phylogenetic relationships among the examined *Cirsium* species.

The *Cirsium* clade was confirmed to be monophyletic (PP = 1.00; Fig. 2A) and further divided into two main subclades: one containing *C. lineare* and the other representing subsect. *Nipponocirsium*. In the present study, two samples of *C. lineare* represented the basal clade of *Cirsium* (Fig. 2A, D, E). Subsect. *Nipponocirsium* was divided into two main subclades: one native to Japan and the other native to Taiwan. In the Japanese clade, *C. kujuense* represented the basal clade and sisters to *C. suffultum* and *C. nipponicum* var. *incomptum*. In the neighbor-net networks (Fig. 2B), these three Japanese taxa exhibited closer genetic distances than Taiwanese clade. By contrast, in the ultrametric Bayesian analysis (Fig. 2D), these Japanese taxa exhibited earlier divergence times. For the Taiwanese clade, the basal clade is *C. pengii*, and sisters to *C. kawakamii* and *C. tatakaense*. In the neighbor-net networks (Fig. 2B), *C. pengii* exhibited greater genetic distances and was positioned closer to *C. kawakamii*. By contrast, *C. tatakaense* displayed higher genetic diversity. Although the *C. tatakaense* sample *SS-3338* was genetically more distant from other *C. tatakaense* samples, it clustered closer to *C. kawakamii*. However, it remained monophyletic with *C. tatakaense*. In the Bayesian analysis, the Taiwanese taxa exhibited greater genetic diversity, but their divergence times were closer (Fig. 2D).

### Species delimitation

All species delimitation methods produced similar results (Fig. 2C), with complete concordance occurring between the SODA and bPTP analyses. Divergence was noted only in the Japanese taxa when the PTP and multithreshold GMYC methods were applied. From a taxonomic perspective, the outgroup (*C. lineare*), *C. kawakamii*, and *C. pengii* were identified as distinct putative species. In phylogenetic analyses, these taxa were resolved as monophyletic groups, with each receiving strong posterior support (Fig. 2A, D, E). Thus, *C. kawakamii* and *C. pengii* can be regarded as two distinct species. According to most analyses, *C. tatakaense* was grouped as a single putative species. However, in the SODA and bPTP analyses, *C. tatakaense_SS-3338* was grouped separately. Thus, although taxonomists generally agree on the species boundary of *C. tatakaense*, the findings of this study indicate that further investigation may be warranted in certain areas.

For the Japanese taxa, clear discrepancies were observed among the results obtained using different methods (Fig. 2C). In the SODA, bPTP, and multithreshold GMYC analyses, the Japanese taxa were grouped into two entities: one comprising *C. suffultum* and *C. nipponicum* var. *incomptum*, and the other comprising *C. kujuense*. By contrast, in the PTP analysis, the Japanese taxa were grouped as a single separate entity. However, in the single-threshold GMYC analysis, each sample was assigned to a separate entity.

### Divergence time

An analysis performed using two CPs revealed that *Gerbera* L. and *Cynara* L. diverged 53.67 (95% CI 49.84–57.50) Myr ago during the Eocene epoch. Furthermore, *Silybum* L. and *Cirsium* diverged 9.8 (95% CI 8.63–10.91) Myr ago during the Miocene epoch (Fig. 3). Furthermore, our findings indicate that *C. lineare* and subsect. *Nipponocirsium* diverged approximately 2.27 (95% CI 1.85–2.69) Myr ago (Fig. 3). The Taiwanese and Japanese taxa diverged approximately 0.74 (95% CI 0.60–0.87) Myr ago. Within the Taiwanese taxa, *C. pengii* diverged from the others approximately 0.47 (95% CI 0.35–0.6) Myr ago, whereas *C. kawakamii* and *C. tatakaense* diverged approximately 0.35 (95% CI 0.26–0.45) Myr ago. This divergence time nearly coincides with the timing of the Mindel glaciation (0.3–0.46 Myr ago) (Lisiecki 2005; Cohen and Gibbard 2019), suggesting that glaciation might have facilitated the divergence of these two taxa. Within the Japanese taxa, *C. kujuense* diverged from the others approximately 0.49 (95% CI 0.21–0.73) Myr ago, whereas *C. suffultum* and *C. nipponicum* var. *incomptum* diverged approximately 0.37 (95% CI 0.08–0.6) Myr ago. Overall, the 95% CI values for the divergence time were broader for the Japanese taxa than for the Taiwanese taxa. This finding indicates greater uncertainty or variability in the divergence timing of the Japanese taxa than in that of the Taiwanese taxa.

### Morphological differences among three Taiwanese taxa of *Cirsium* subsect. *Nipponocirsium*

**Fig. 4:**
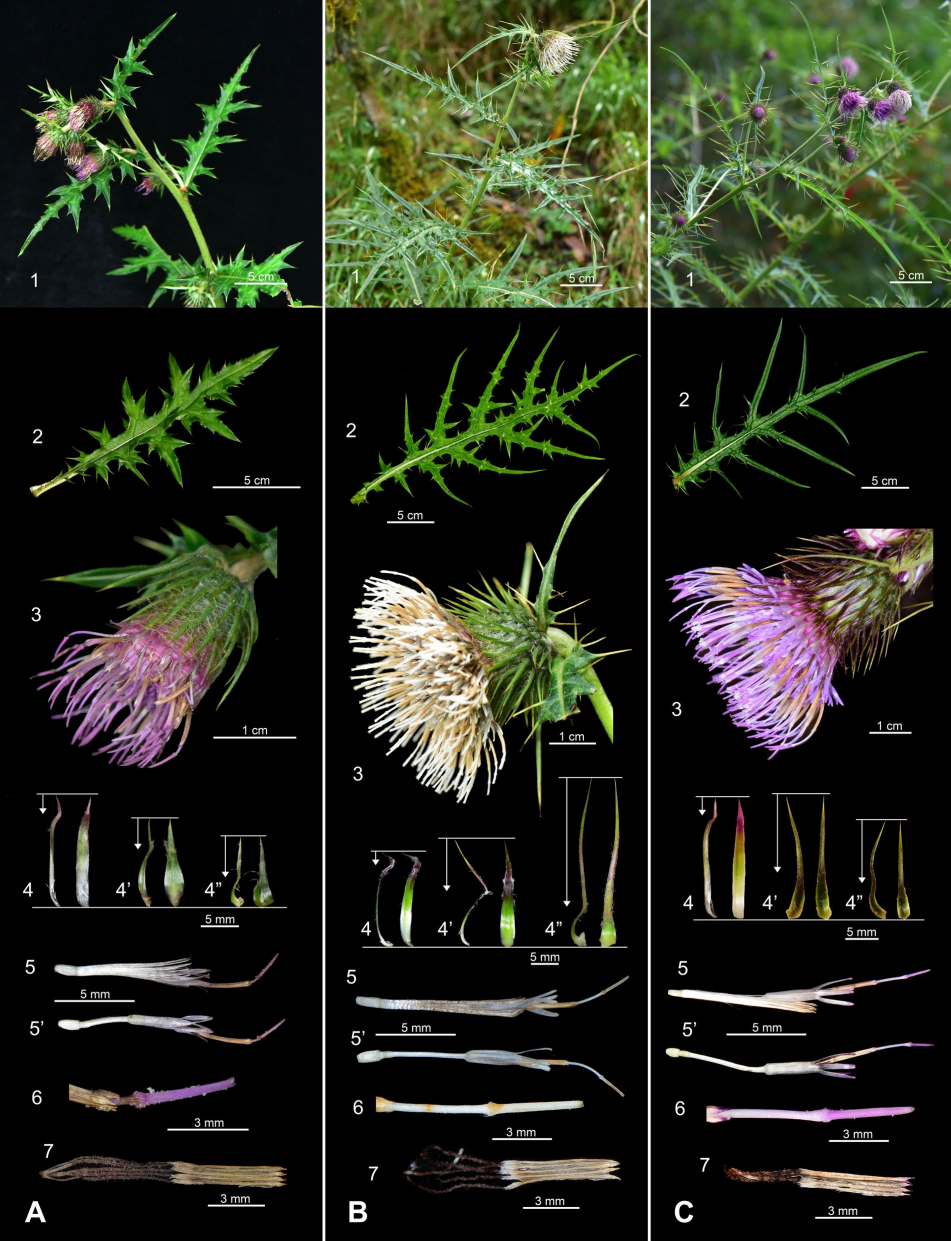
Comparison of the morphological characters of *Cirsium* Mill. subsect. *Nipponocirsium* Kitam. of Taiwan. **A:** *C. pengii* Y.H.Tseng, P.C.Liao & Chih Y.Chang; **B:** *C. kawakamii* Hayata; **C:** *C. tatakaense* Y.H.Tseng & Chih Y.Chang. **1:** habit; **2:** leaf; **3:** Capitula; **4:** inner phyllary; **4’:** middle phyllary; **4″:** outer phyllary; **5:** floret; **5’:** floret (pappus removed); **6:** style branches; **7:** synantherous stamen

The average leaf width of *C. pengii* (6.46 cm) was significantly smaller than that of *C. kawakamii* (15.31 cm) and *C. tatakaense* (17.99 cm). Similarly, the lobe length of *C. pengii* (2.79 cm) was significantly smaller than that of *C. kawakamii* (8.57 cm) and *C. tatakaense* (9.08 cm). The leaf shape of *C. pengii* ranged from narrowly elliptic to elliptic, with a pinnatipartite margin (Fig. 4A2). By contrast, in *C. kawakamii* and *C. tatakaense*, the leaf shape ranged from elliptic to broadly elliptic, with a pinnatisect or bipinnatisect margin (Fig. 4A2, B2). *Cirsium tatakaense* generally has the slenderest lobes of the Taiwanese taxa (Chang et al. 2019). However, some *C. tatakaense* populations at the southern boundary of its distribution exhibit broader lobes resembling those of *C. kawakamii* (see Discussion).

The involucre widths of *C. kawakamii* and *C. tatakaense* were 2.03 and 2.17 cm, respectively (Fig. 4B3, C3); both values were significantly larger than that of *C. pengii* (1.15 cm) (Fig. 4A3). Notably, *C. pengii* has a pot-shaped involucre, characterized by a narrower width at the upper portion than at the base (Fig. 4A3). By contrast, *C. kawakamii* and *C. tatakaense* have a bowl-shaped involucre, characterized by a wider width at the upper portion than at the base (Fig. 4B3, C3). In this study, the phyllaries of *C. kawakamii* (approximately 1.80 cm, Fig. 4B4) were significantly longer than those of other taxa; however, no significant difference was observed between the phyllary lengths of *C. pengii* (0.78 cm, Fig. 4A4) and *C. tatakaense* (1.04 cm, Fig. 4C4). The total phyllary count was higher for *C. tatakaense* (n = 154) than for *C. pengii* (n = 113) and *C. kawakamii* (n = 99; Table 1); this finding is consistent with those of Chang et al. (2019). Regarding corolla color, both *C. pengii* and *C. tatakaense* exhibited bluish-purple corollas (Fig. 4A3, 5; C3, 5), whereas *C. kawakamii* had a white corolla (Fig. 4B3, 5). Furthermore, *C. kawakamii* had significantly longer florets, corolla lobes (Fig. 4B5), and synanthers (Fig. 4B7) than did the other two species. The floret count was higher for *C. tatakaense* (n = 254) than for *C. pengii* (n = 120) and *C. kawakamii* (n = 138; Table 1).

**Table 1:** Comparison of 3 taxa of *Cirsium* Mill. subsect. *Nipponocirsium* Kitam. in Taiwan

Taxon		*C. pengii*	*C. kawakamii*	*C. tatakaense*
Cauline leaves	Size (cm)	14.02 ± 6.77^a^ × 6.46 ± 2.65^a^	26.35 ± 9.78^a^ × 15.31 ± 5.27^b^	25.77 ± 11.21^a^ × 17.99 ± 8.79^b^
	Shape	Narrowly elliptic to elliptic	Elliptic to broadly elliptic	Elliptic to broadly elliptic
	Aspect ratio	2.13 ± 0.21^b^	1.71 ± 0.15^a^	1.47 ± 0.26^a^
	Margin	Pinnatipartite	Pinnatisect or bipinnatisect	Pinnatisect or bipinnatisect
	Lobe Size (cm)	2.79 ± 1.18^a^ × 1.25 ± 0.50^a^	8.57 ± 2.97^b^ × 1.77 ± 0.41^a^	9.08 ± 3.62^b^ × 1.18 ± 0.69^a^
	Pair of leaflobes	5 ± 1^a^	7 ± 1^a^	6 ± 2^a^
Capitula	Size (cm)	2.64 ± 0.38^a^ × 1.15 ± 0.04^a^	4.78 ± 0.98^ab^ × 2.03 ± 0.25^b^	4.82 ± 1.73^b^ × 2.17 ± 0.70^b^
	Involucre shape	Pot-shaped (upper width < base)	Bowl-shaped (upper width ≥ base)	Bowl-shaped (upper width ≥ base)
Phyllary	length (cm)	0.78 ± 0.13^a^	1.80 ± 0.04^b^	1.04 ± 0.35^a^
	protrusion (mm)	0.51 ± 0.09^a^	1.29 ± 0.93^a^	0.68 ± 0.30^a^
	number	113 ± 2^a^	99 ± 21^a^	154 ± 32^b^
Floret	length (cm)	2.81 ± 0.18^a^	3.71 ± 0.13^b^	2.85 ± 0.30^a^
	Corolla color	Bluish-purple	White	Bluish-purple
	Corolla lobe size (mm)	4.46 ± 0.28^a^ × 0.86 ± 0.06^c^	5.75 ± 1.07^b^ × 0.70 ± 0.05^b^	4.09 ± 0.64^a^ × 0.52 ± 0.12^a^
	synanther length (mm)	6.81 ± 0.15^a^	8.91 ± 2.10^a^	6.31 ± 0.87^b^
	number	120 ± 5^a^	138 ± 64^a^	254 ± 71^b^
Pollen	Size (P/E, μm)	52.10 ± 3.66^b^/46.11 ± 2.12^a^	42.27 ± 6.49^a^/47.64 ± 2.09^a^	39.84 ± 3.16^a^/41.80 ± 3.40^a^
	PE ratio	1.13 ± 0.08^b^	0.95 ± 0.05^a^	0.96 ± 0.08^a^
	Pollen spine size (μm)	3.75 ± 0.52^b^ × 5.12 ± 0.49^b^	2.86 ± 0.74^a^ × 3.86 ± 0.72^a^	3.90 ± 0.78^b^ × 6.09 ± 1.25^c^
Chromosome number		2*n* = 2*x* = 32 (this study)	2*n* = 4*x* = 64 (Chang et al. 2019 and this study)	2*n* = 4*x* = 64 (Chang et al. 2019)
Distribution		Endemic to Taiwan; only known in the Lala Mountain area at an altitude of about 1700 m	Endemic to Taiwan; gullies and valleys at 2500–3500 m alt. central-northern Taiwan (Chang et al. 2019)	Endemic to Taiwan; open areas of fog forests at 2000−3000 m alt. central-southern Taiwan (Chang et al. 2019)

^abc^Means in a row without a common superscript letter different (*p* ≤ 0.05; Tukey’s HSD test)

### Differences in pollen morphology among three Taiwanese taxa of *Cirsium* subsect. *Nipponocirsium*

**Fig. 5:**
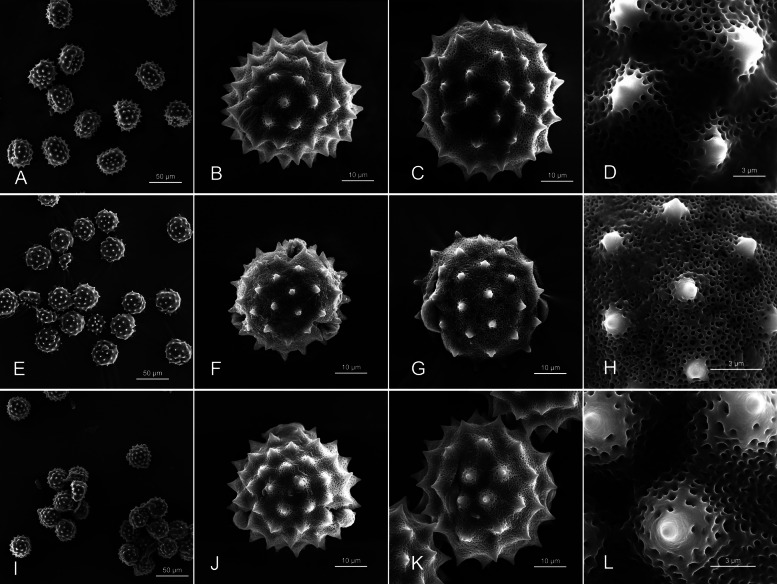
Comparison of the pollen morphology of *Cirsium* Mill. subsect. *Nipponocirsium* Kitam. in Taiwan. **A–D:** *C. pengii* Y.H.Tseng, P.C.Liao & Chih Y.Chang; **E–H**: *C. kawakamii* Hayata; **I–L:** *C. tatakaense* Y.H.Tseng & Chih Y.Chang. **A**, **E**, **I:** pollen grain; **B**, **F**, **J:** polar view; **C**, **G**, **K:** equatorial view; **D**, **H**, **L**: enlarged of equatorial view

The pollen grains were tricolporate, nearly spheroidal, and medium-sized; had a microreticulate surface; and featured densely packed pollen spines (Fig. 5). The polar axial length of pollen grains was significantly the longest in *C. pengii* (52.10 μm); no significant difference was observed in this parameter between *C. kawakamii* (42.27 μm) and *C. tatakaense* (39.84 μm). Furthermore, the P/E ratio was significantly the highest in *C. pengii* (1.13; slightly prolate shape); no significant difference was noted in this parameter between *C. kawakamii* (0.95; spherical shape) and *C. tatakaense* (0.96; spherical shape). Furthermore, the size of pollen spines was significantly the largest in *C. tatakaense* (length × width: 3.90 μm × 6.09 μm) and then in *C. pengii* (3.75 μm × 5.12 μm) and *C. kawakamii* (2.86 μm × 3.86 μm; Fig. 4; Table 1).

### Results of Chromosome number analysis

**Fig. 6:**
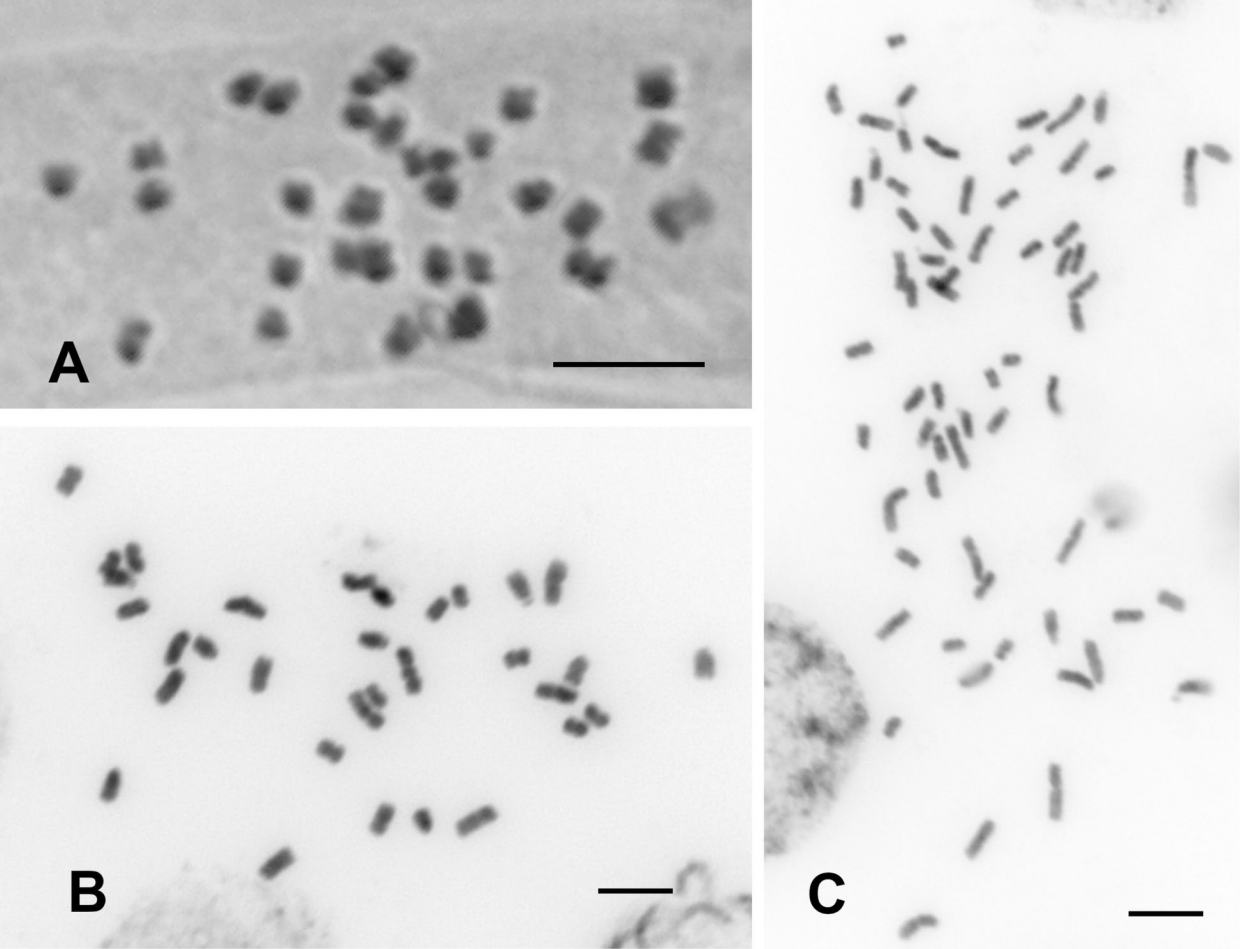
Chromosome number analysis of *Cirsium* Mill. subsect. *Nipponocirsium* in Taiwan. **A–B**: *C. pengii* Y.H.Tseng, P.C.Liao & Chih Y.Chang; **C**: *C. kawakamii* Hayata. **A**: the squash method; **B–C**: the flame-drying method, the images were processed as negatives to enhance chromosome visualization. Scale bars = 5 μm

We found that *C. pengii* is a diploid species with a chromosome number of 2*n* = 2*x* = 32 (Fig. 6A, B), while *C. kawakamii* are tetraploid species with a chromosome number of 2*n* = 4*x* = 64 (Fig. 6C).

## Discussion

### Polyploidization as a potential mechanism for radiation evolution of *Cirsium* subsect. *Nipponocirsium*

The most common diploid chromosome number in *Cirsium* is 2*n* = 2*x* = 34 (*x* = 17) (Moore and Franicton 1963; Hsu 1970; Peng and Hsu 1978; Masukawa et al. 1990; Bureš et al. 2004; Özcan et al. 2008, 2011; Nouroozi et al. 2010; Polat et al. 2018), which also represents ancestral (Bureš et al. 2004; Mota et al. 2016; Ackerfield et al. 2020a). This number likely derived from a paleopolyploid ancestor (2*n* = 4*x* = 36) through descending dysploidy (Mota et al. 2016). Subsect. *Nipponocirsium* members are typically tetraploid, with chromosome counts of 2*n* = 68 in Japan (Kadota 1995; Kadota and Miura 2014, 2021) and 2*n* = 64 in Taiwan (Chang et al. 2019), indicating divergence between these regions.

*Cirsium pengii* exhibited a chromosome number of 2*n* = 32 (Fig. 6) and was positioned as the basal group of the Taiwan clade in phylogenetic analysis (Fig. 2). This suggests its ancestor underwent descending dysploidy to *x* = 16, followed by polyploidization events, potentially giving rise to *C. kawakamii* and *C. tatakaense*. Thus, *C. pengii* may represent a primitive karyotype, supporting the role of polyploidy in speciation within subsect. *Nipponocirsium*.

Bureš et al. (2023) reported that descending dysploidy and polyploidization are relatively common phenomena in the subtribe Carduinae, particularly in *Carduus* and the North American *Cirsium* taxa, where these processes indicate strong phylogenetic signals. Polyploidy may facilitate reproductive isolation and nonadaptive radiation (Soltis et al. 2009; Gorelick and Olson 2013; Cannon et al. 2015; Van de Peer et al. 2017). Although polyploidy is not random and appears to be correlated with periods of environmental upheaval, it may increase the adaptive potential of cells and organisms under stressful conditions (Van de Peer et al. 2017). Global climate change may promote polyploidization events in plants (Cai et al. 2019; Sessa 2019; Levin 2019; Zhang et al. 2020); this is because polyploids often exhibit broader ranges of ecological tolerance and invasiveness than do their diploid relatives (Levin 2019). For instance, polyploidization events during the Mindel glaciation (~ 0.26–0.6 Myr ago) may have driven karyotype evolution in Taiwanese taxa (Fig. 3).

The Japanese taxa of subsect. *Nipponocirsium* typically have a chromosome number of 2*n* = 4*x* = 68 (Kadota 1995, 2006; Kadota and Miura 2014, 2021). Kadota and Miura (2021) reported that some species with appearances similar to that of subsect. *Nipponocirsium*, for example, *C. kubikialpicola* Kadota (subsect. *Praticola* Kadota), *C. rengehydrophilum* Kadota (subsect. *Reflexae* (Kitam.) Kadota), and very few *C. nipponicum* var. *incomptum* (Kitamuta et al. 1957), have diploid karyotypes (2*n* = 2*x* = 34). However, a complete phylogenetic analysis in Japan is needed for further insights.

### Recurrent glacial periods might have facilitated the divergence of *Cirsium* subsect. *Nipponocirsium* taxa in Taiwan

Taiwan’s unique geographic and climatic conditions render it an ideal natural laboratory for studying species divergence and ecological adaptation. Located at the intersection of the Paleotropical and Holarctic floristic kingdom (Good 1964; Takhtajan 1986), Taiwan has several peaks exceeding 3500 m. The highest peak, Mt. Yushan, is 3952 m tall, and therefore, Taiwan has the fourth-highest island mountain in the world (Chen 1980; Wang et al. 2009a). Because of its geographic conditions, Taiwan’s fauna and flora have frequently been affected by recurring glacial periods (Tsukada 1967; Liew et al. 1998). During glacial epochs, reductions in sea levels result in the formation of land bridges on continental shelves, facilitating species migration from surrounding landmasses. By contrast, during interglacial phases, rising sea levels submerge these land bridges (Rohling et al. 2008), isolating populations that have migrated to Taiwan from their source populations (Huang 1987, 2011; Shen 1996; Wang et al. 2003).

Glacial epochs are characterized by a considerable decline in temperature and exert a bottleneck effect on biological populations. However, such Taiwan’s north–south oriented mountain ranges and diverse ecological niches provide critical refuges for organisms during these periods (Hsieh and Shen 1994; Hsieh et al. 1994; Anthelme et al. 2008; Garrick 2011). During interglacial periods, warmer climates facilitate the expansion of organisms from these refuges, often leading to colonization of high altitudes by species intolerant to elevated temperatures. Examples of such species are *Cunninghamia* R. Br. (Hwang et al. 2003), *Juniperus* L. (Huang et al. 2016), and *Anourosorex* (Yuan et al. 2006). Therefore, recurring glaciation exerts selective pressures on organisms, driving species divergence within the island. This process is reflected in Taiwan’s high proportion of endemic plant species (Hsieh 2002).

In the divergence time analysis (Fig. 3), we investigated the potential association between glaciation events and the divergence time of subsect. *Nipponocirsium*. These Taiwanese and Japanese taxa likely diverged 95% CI 0.60–0.87 Myr ago, primarily during interglacial periods between the Pre-Pastonian and Günz glaciation periods. Kim et al. (2023) suggested that the ancestral origin of the core member of subsect. *Nipponocirsium*, *C. nipponicum*, can be traced back to the northern regions of the Eurasian continent. Therefore, the divergence of the Taiwanese and Japanese taxa might have been driven by population isolation caused by rising sea levels during interglacial periods.

Among the Taiwanese taxa, *C. pengii* exhibited the earliest divergence, estimated at 95% CI 0.35–0.60 Myr ago, primarily between the Günz and Mindel glaciation periods. *Cirsium pengii* is characterized by its low-elevation distribution and diploid ploidy level (2*n*), in contrast to the high-elevation distribution and tetraploid ploidy level (4*n*) of *C. kawakamii* and *C. tatakaense* (Chang et al. 2019). The time of divergence between *C. kawakamii* and *C. tatakaense* was approximately 95% CI 0.26–0.45 Myr ago, closely aligning with the timing of the Mindel glaciation (0.3–0.46 Myr) (Lisiecki 2005; Cohen and Gibbard 2019). Different distribution of these two taxa in Taiwan, with *C. kawakamii* inhabiting the central-northern mountainous regions and *C. tatakaense* found in the southern mountainous regions (Chang et al. 2019). Suggesting that these taxa retreated to refugia in the northern and southern regions, respectively, during the Mindel glaciation.

For the Japanese taxa, the divergence time estimates revealed broad 95% CIs including multiple glacial and interglacial periods. This variability increased the difficulty of identifying specific factors affecting their divergence; further research is required on this topic. In summary, Taiwan’s complex geographic and climatic history, particularly its recurring glacial and interglacial cycles, might have shaped the island’s biodiversity. Our findings clarify how climatic fluctuations may have driven species migration, isolation, and divergence in *Cirsium*, contributing to the high levels of endemism observed in modern times.

### Genetic complexity and species divergence: Insights from DensiTree and species delimitation analysis

The DensiTree visualization presented in Fig. 3 reveals several conflicting events that are not apparent in the phylogenetic tree. These events might have resulted from gene flow, hybridization, or incomplete lineage sorting (Joly et al. 2009). A complex branching structure was observed among the three Japanese taxa. The neighbor-net networks depicted in Fig. 2B revealed relatively close genetic distances. Several studies (Bureš et al. 2004, 2010; Nouroozi et al. 2010; Mameli et al. 2014) have reported incomplete reproductive isolation and frequent interspecific hybridization within *Cirsium*. Such hybridization events can blur species boundaries because of the admixture of parental genomes (Givnish 2010; Wagner et al. 2020). Our species delimitation analysis revealed clear differentiation among the three Japanese taxa (Fig. 2C), with many nodes in their phylogenetic relationships receiving strong support (Fig. 2A, D, E). On the basis of recent classifications (Kitamuta et al. 1957; Kadota 1995, 2006, 2008), the Japanese taxa were regarded as three distinct species; this classification aligns with the results of only the single-threshold GMYC analysis. However, in our study, each species was represented by only a single sample. Therefore, for a more objective taxonomic classification of the Japanese taxa, the sampling range should be expanded in future studies.

Regarding the Taiwanese taxa, some weak gene flow signals were detected between *C. kawakamii* and the other taxa. Specifically, this gene flow was observed between the sample *NG-3861* and *C. tatakaense* and between the sample *CL-3544* and *C. pengii*. This finding aligns with the geographic distribution of these taxa: The distribution of *C. kawakamii* in Taiwan is roughly between the distribution of *C. pengii* and that of *C. tatakaense*. The relatively limited sympatric distribution of these taxa might have led to the weak signal, indicating that gene flow events likely occur at a low frequency. Our species delimitation analysis revealed well-defined clustering (Fig. 2C), with major nodes receiving strong support (Fig. 2A, D, E). Given the potential sympatric distribution of these taxa, species divergence might have occurred in the presence of gene flow. Wu (2001) indicated that the fundamental unit of adaptation is a group of interacting genes; because of the hitchhiking effect, genomic regions resulting from reproductive isolation gradually expand, ultimately leading to speciation. Chang et al. (2022) provided evidence of divergence caused by gene flow in *Cycas* L. populations across the East Asian Island arc. Similarly, Seehausen (2004) suggested that favorable ecological conditions for hybridization can accelerate species diversity through adaptive radiation.

### Genetic diversity and morphological variations in *C. tatakaense*

The neighbor-net network (Fig. 2B) revealed that *C. tatakaense* exhibited higher genetic diversity than did the other species. Notably, the *SS-3338* sample exhibited a closer genetic affinity to *C. kawakamii* but remained monophyletic with other *C. tatakaense* samples (Fig. 2). In the species delimitation analysis (Fig. 2C), most methods (PTP, multithreshold GMYC, and single-threshold GMYC) classified the broad *C. tatakaense* cluster as a single species. However, SODA and bPTP treated *SS-3338* as a different species. This inconsistency might have resulted from the insufficient sample size.

Morphologically, *SS-3338* exhibited broader leaf lobes than did the holotypic form of *C. tatakaense*. However, no significant differences were observed in other external characteristics or pollen morphology. Whether hybridization occurs between *SS-3338* and other taxa remains uncertain because they often have a sympatric distribution. Thus, although *SS-3338* may currently be regarded as a member of *C. tatakaense*, its taxonomic status warrants further investigation.

### Taxonomic status of the unknown *Cirsium* taxon

The unknown taxon exhibited distinct morphological characteristics and a unique karyotype compared with other species within subsect. *Nipponocirsium* in Taiwan. A detailed comparison of the morphological characteristics of *subsect. Nipponocirsium* from other regions is provided in Supp. table 4. The unknown taxon had narrower and smaller leaves with shorter leaf lobes than did the other taxa. Its involucre was pot-shaped, with the upper portion being narrower than the base. The unknown taxon featured a bluish-purple corolla (Fig. 4A3, 5–6), which differs from the white corolla of its sister species *C. kawakamii* (Fig. 4B3, 5–6) but is similar to that of *C. tatakaense* (Fig. 4C3, 5–6). However, the unknown taxon is geographically distinct from both species, thriving at an altitude of approximately 1700 m in northern Taiwan. By contrast, *C. kawakamii* and *C. tatakaense* are found at altitudes of > 2000 m (Peng 1998) in central-northern and central-southern Taiwan, respectively (Chang et al. 2019) (Table 1).

The unknown taxon exhibited prolate pollen grains, whereas both *C. kawakamii* and *C. tatakaense* had spherical pollen grains. *Cirsium tatakaense* had the largest pollen grain and pollen spine, whereas *C. kawakamii* had the smallest one. The unknown taxon exhibited intermediate pollen grain and spine sizes, with the sizes falling between those of the other two species (Fig. 5; Table 1). Plant size was generally larger for *C. kawakamii* (Fig. 4B1–5) and *C. tatakaense* (Fig. 4C1–5) than for *C. pengii* (Fig. 5A1–5; Table 1). Additionally, the chromosome number of the unknown taxon was 2*n* = 2*x* = 32, differing from those of the known species of subsect. *Nipponocirsium* in Taiwan (2*n* = 4*x* = 64) (Chang et al. 2019) and Japan (2*n* = 4*x* = 68) (Kadota 1995; Kadota and Miura 2014, 2021). Polyploidization promotes plant growth, thereby enabling plants to rapidly adapt to diverse environments (Bamford and Winkler 1941; Kulkarni and Borse 2010; Van de Peer et al. 2021). In summary, the unknown taxon is clearly different from known congeners. Therefore, we propose formal classifying the unknown taxon as a new species, *C. pengii*.

### Key to the infrageneric classification of native *Cirsium*, including *C. pengii* and its closely related taxa in Taiwan

1. Biennial herb; involucre tube-shaped (length twice the width); corolla lobes < 2.5 mm long.sect. I. *Pseudoeriolepis*, incl. *C. ferum*
2. Perennial herb; involucre pot or bowl-shaped (length approximates width), corolla lobes > 2.5 mm long:
  2. Leaves linear, abaxial surface densely cobwebby; apical parts of inner phyllaries inflated, obtuse; corolla lobes longer than the inflated part of corolla tube…sect. II. *Spanioptilon*, incl. *C. lineare*.
3. Leaves widely, narrowly elliptic to oblance-ovate, abaxial surface glabrous, pubescent, hirsute or cobwebby; apical parts of inner phyllaries acute or acuminate; corolla lobes shorter or as long as the inflated part of the corolla tube…sect. III. *Onotrophe.*
  3. Leaves both basal rosette and cauline, only rosette leaves petiolate, cauline leaves sessile, pubescent, hirsute or cobwebby:
    4. Mature capitula erect; apical parts of prominent phyllaries spine-like (conical); corolla lobes shorter than the inflated parts of the corolla tube…Subsect. III. *Sinocirsium*, incl. *C. japonicum* var. *australe.*
    5. Mature capitula erect or nodding; apical parts of prominent phyllaries flat shape; corolla lobes as long as the inflated parts of the corolla…tubeSubsect. IV. *Australicirsium*, incl. *C. arisanense.*

3. Leaves all cauline, rarely both basal rosette and cauline, all petiolate, glabrous or less cobwebby:
  5. Mature capitula erect; leaves both basal rosette and cauline; corollas light purple; stems both cauline and rhizomatous…subsect. I. *Arenicola*, incl. *C. morii.*
  6. Mature capitula nodding; leaves all cauline, basal rosette leaves absent; corollas white or bluish purple; stems only cauline (subsect. II. *Nipponocirsium*):
  7. Involucre pot-shaped; leaves narrowly elliptic, lobes < 4 cm long; corollas bluish purple…4. *C. pengii.*
  8. Involucre bowl-shaped; leaves widely elliptic, lobes > 5 cm; corollas white or bluish purple:
  7. Corollas white; leaves pinnatisect or bipinnatisect, lobes > 15 mm wide.…5. *C. kawakamii.*
  8. Corollas bluish purple; leaves mainly pinnatisect, lobes < 12 mm wide…6. *C. tatakaense.*

### Taxonomic treatment

**Fig. 7:**
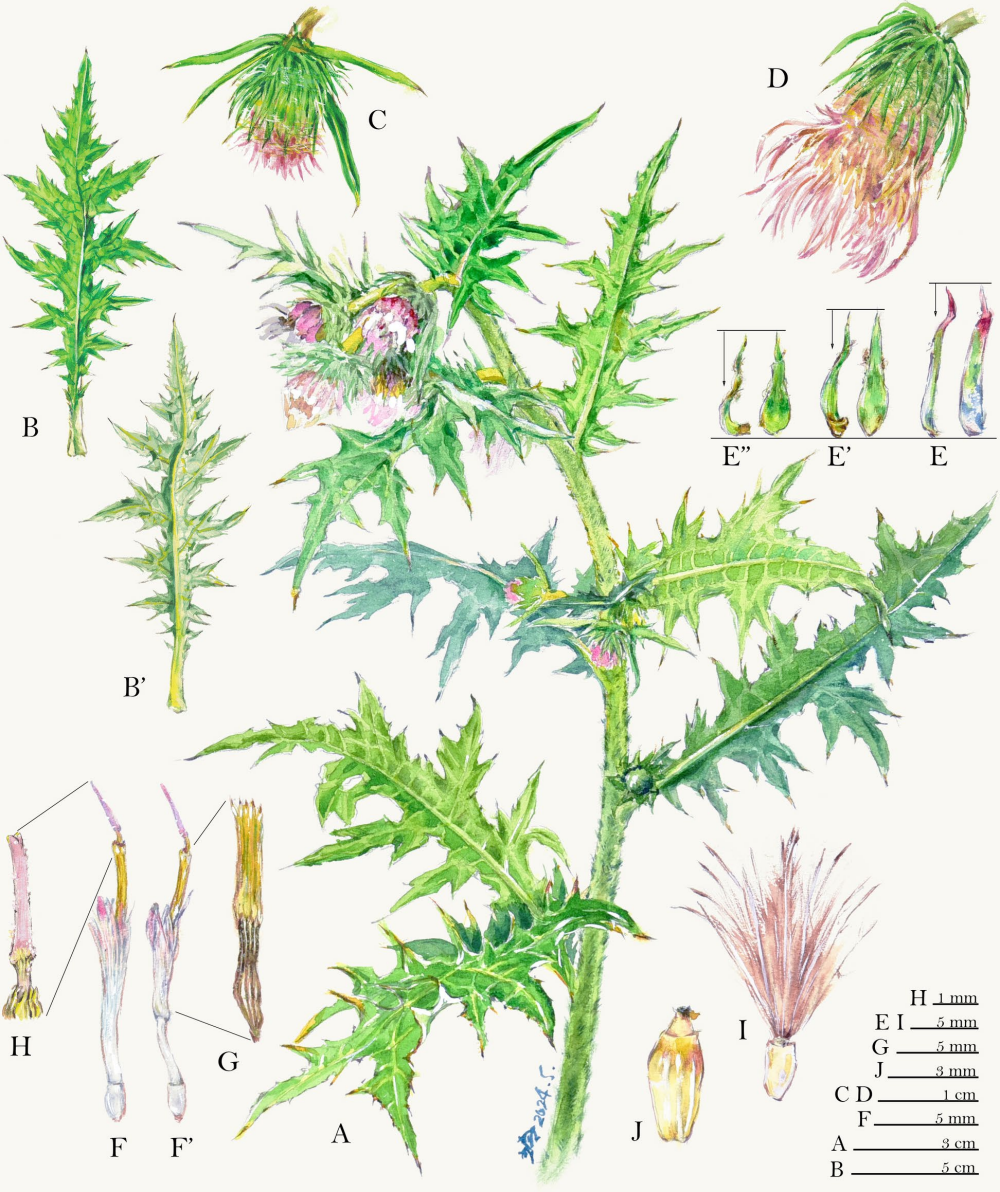
Color illustration of* Cirsium pengii* Y.H.Tseng, P.C.Liao & Chih Y.Chang. **A**: habit; **B**: leaf adaxial; **B’**: leaf abaxial; **C**: buds; **D**: capitula; **E–E**”: inner to outer phyllaries; right: abaxial view; left: side view; **F**: floret; **F**’: floret (remove pappus); **G**: synantherous stamen; **H**: style branches;** I**: achene with pappus; **J:** achene. Voucher: *C.Y. Chang 4310* (TCF)

**Cirsium pengii** Y.H.Tseng, P.C.Liao & Chih Y.Chang, *sp. nov.* (Fig. 4A, Fig. 7).

**Diagnosis:** Differs from *C. kawakamii* and *C. tatakaense* on the basis of its narrower and smaller leaves, shorter leaf lobes, and narrower involucre. Differs from *C. kawakamii* in having a bluish-purple corolla (vs. white).

**Fig. 8:**
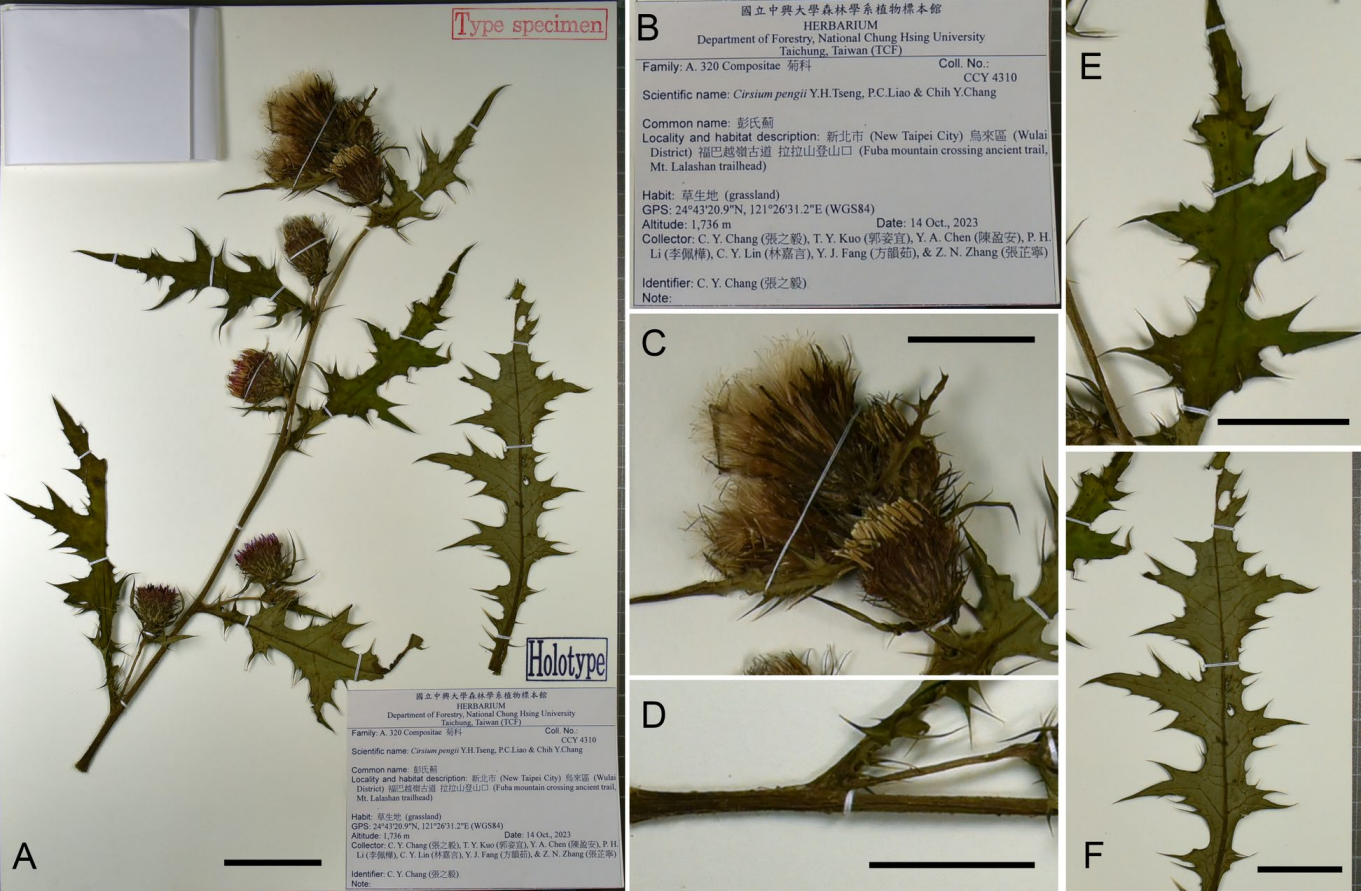
Holotype of *Cirsium pengii* Y.H.Tseng, P.C.Liao & Chih Y.Chang. **A**: full specimens; **B**: label; **C:** capitula; **D:** stem; **E**: leaf adaxial surface; **F**: leaf abaxial surface

**Type:** TAIWAN. New Taipei City, Wulai District, Mt. Lalashan, alt. 1736 m, 24°43′20.9"N, 121°26′31.2"E, 14 Oct. 2023, *C.Y. Chang 4310* (holotype: TCF, Fig. 8; isotype: TNM, HAST).

**Description:** Perennial herbs, stems 0.2–1.2 m tall, without rosette leaves. Leaves alternate, pinnatipartite, 6.5–26.5 cm long, 3.3–10.8 cm wide, glabrous, narrowly elliptic to elliptic, base truncate to cuneate, apex caudate; pinnae 5–8 pairs, 1.5–4.6 cm long, 0.6–2.2 cm wide; space between pinnae U–shaped, distance 2–2.5 cm. Capitula solitary or arranged into corymb or panicle, mature capitula nodding, 2.3–3.2 cm long, 1.1–1.2 cm wide; involucre pot–shaped, lacking abaxial appendages, 0.7–1.5 cm long, 1.5–2.2 mm wide, protrusion 3–6.1 mm, inner phyllaries acute apically, bluish purple, outer phyllaries spinose apically, green. Florets with bluish purple corolla, 2.5–3 cm long, corolla lobes 4.2–4.9 mm long, 0.8–0.9 mm wide; 5 synantherous stamens, detached filaments with irregular protuberances, basal caudate extensions, bluish purple, anthers 6.6–7 mm long, filaments 8.1–9.3 mm long. Stigmas bifid apically, style 2.3–2.8 cm long, ovaries 2.5–2.9 mm long. Achenes oblong, base acute, apex truncate, beige, 4.5–5.1 mm long, 1.7–2.1 mm wide, apical with beak. Pappus 1.6 cm long forming basal ring, easily shed.

**Chinese name:** Peng-shih-ji (彭氏薊).

**Chromosome number:** 2*n* = 2*x* = 32.

**Distribution and habitat:** Endemic to Taiwan, this species is found in the Mt. Lalashan area at an altitude of approximately 1700 m, within the *Quercus* zone and the northwest inland region, as classified by Su (1984, 1985). Common companion species include *Miscanthus sinensis* Andersson var. *glaber* (Nakai) J.T. Lee (Poaceae), *Microstegium biaristatum* (Steud.) Keng (Poaceae), *Eurya crenatifolia* (Yamamoto) Kobuski (Pentaphylacaceae), *Callicarpa formosana* Rolfe (Lamiaceae), and *Diplazium mettenianum* (Miq.) C. Chr. (Athyriaceae).

**Phenology:** Flowering between August and November and fruiting between September and December.

**Etymology:** The species epithet “*pengii*” was selected to memorialize Dr. Ching-I Peng (彭鏡毅) (1950–2018), an “outstanding scientist and mentor with a remarkable legacy” (Chung 2020), who was also the first collector of this species.

**Pollen morphology:** Pollen grains are tricolporate, prolate, micro-reticulate and 45.1 − 57.4 × 42.9 − 50.0 μm in size (P/E ratio: 1.0 − 1.2). The surface is densely covered with spines that are 2.9 − 4.5 μm long and 3.8 − 6.0 μm wide (at the base). The distance between two spines is 9.4 − 12.5 μm (Fig. 5A–D).

**Conservation status:** To the best of our knowledge, *C. pengii* is found only in the open regions of the forest margin in the Mt. Lalashan area, at an altitude of approximately 1700 m. This is the only type of locality known thus far. Therefore, following the criteria outlined by the IUCN (2019), we regard this taxon as Critically Endangered (CR B2a(iii); C2a(i; ii); D). Naming this species is crucial for its conservation and understanding its evolutionary importance; in addition, it can help in clearly defining the substantial conservation value of this species.

**Additional specimens examined:** TAIWAN. **New Taipei City:** Wulai District, Mt. Lalashan, *C. I Peng 14,628* (HAST17858!, Fig. 1A).

## Conclusion

We elucidated the phylogenetic relationships, divergence time, and chromosomal evolution of *Cirsium* sect. *Onotrophe* subsect. *Nipponocirsium.* Chromosome analyses indicated the pivotal roles of polyploidization and dysploidy in the evolutionary radiation of *Cirsium* taxa. *Cirsium pengii*, a new diploid species that differs from the known tetraploid species *C. kawakamii* and *C. tatakaense*, indicating chromosomal variations as a key factor in speciation. These findings demonstrate the importance of integrating transcriptomic analysis with traditional morphological and cytological methods to understand complex mechanisms underlying speciation and chromosomal evolution. This comprehensive framework can improve the understanding of the genetic diversity and adaptive mechanisms in *Cirsium* sect. *Onotrophe* subsect. *Nipponocirsium* and guide further investigations into the evolutionary biology of this ecologically important group.

## Supplementary Information

Supplementary material 1.

## Data Availability

Raw sequence reads have been submitted to the Sequence Read Archive (SRA) of the National Center for Biotechnology Information (NCBI) under BioProject ID PRJNA1158676. These data are available in read-only format at the following link: https://dataview.ncbi.nlm.nih.gov/object/PRJNA1158676?reviewer=1epu9k327mlidaifv86jt3qgdc.
