# Correction: Chromosome number variation and phylogenetic divergence of East Asian *Cirsium* sect. *Onotrophe* subsect. *Nipponocirsium* (Compositae), with a new species from Taiwan

**DOI:** 10.1186/s40529-025-00458-y

**Published:** 2025-03-27

**Authors:** Chih-Yi Chang, Pei-Chun Liao, Hsy-Yu Tzeng, Junko Kusumi, Zhi-Hui Su, Yen-Hsueh Tseng

**Affiliations:** 1 Taiwan Forestry Research Institute, No. 53, Nanhai Rd., Zhongzheng Dist, Taipei, 10066 Taiwan; 2 School of Life Science, National Taiwan Normal University, No. 88, Sec. 4, Ting-Chow Rd., Wenshan Dist. 116, Taipei, Taiwan; 3 Department of Forestry, National Chung-Hsing University, No. 145, Hsing-Ta Rd, Taichung, 402 Taiwan; 4 Department of Environmental Changes, Faculty of Social and Cultural Studies, Kyushu University, Fukuoka, 819-0395 Japan; 5 JT Biohistory Research Hall, Takatsuki, 569-1125 Osaka Japan; 6 Department of Biological Sciences, Graduate School of Science, Osaka University, Osaka, 560-0043 Japan

**Correction to: Botanical Studies (2025) 66:8**

10.1186/s40529-025-00454-2

Following publication of the original article (Chang et al. 2025), the authors reported that the second author’s name was mistakenly listed as “Pei-Chung Liao”. The correct spelling should be “Pei-Chun Liao.”

The correct author’s name has been provided in this Correction.

The original article (Chang et al. 2025) has been corrected.
