# Supplementary material for: Chromosome number variation and phylogenetic divergence of East Asian Cirsium sect. Onotrophe subsect. Nipponocirsium (Compositae), with a new species from Taiwan
Source: Bot Stud. 2025 Feb 14;66:8. doi: 10.1186/s40529-025-00454-2 (PMC11828781; doi:10.1186/s40529-025-00454-2)
Supplement: Supplementary file 1 — Supplementary material 1. [file 40529_2025_454_MOESM1_ESM.docx]

Supp. table 1. Bioinformation and voucher of phylogeny reconstruction

| **Taxon** | **Raw data** | | **Trimmed** | | **Trinity (C/D, %)** | **cd-hit-est (C/D, %)** | **Collecting location** | **Coordinate** | **Altitude (m)** | **Voucher** |
| --- | --- | --- | --- | --- | --- | --- | --- | --- | --- | --- |
|  | **Number of reads** | **GC%** | **Read (M)** | **GC%** |  |  |  |  |  |  |
| *C. lineare* | 62,493,916 | 45 | 25.7 | 45 | 82.8/37.1 | 82.5/13.2 | TAIWAN. Miaoli County: Xihu | 24°29'N, 120°45'E | 173 | *ccy3446* |
|  | 37,079,400 | 47 | 10.9 | 45 | 44.3/15.1 | 44.0/3.3 | TAIWAN. Miaoli County: Tongxiao | 24°26'N, 120°41'E | 81 | *ccy2770* |
| *C. tatakaense* | 65,271,584 | 45 | 27.4 | 43 | 87.1/40.4 | 86.7/17.9 | TAIWAN. Nantou County: Luanantang | 23°42'N, 120°57'E | 2,476 | *ccy4022* |
|  | 41,746,414 | 50 | 6.4 | 45 | 15.1/3.1 | 15.0/0.8 | TAIWAN. Chiayi County: Mt. Yushan | 23°28'N, 120°54'E | 2,866 | *ccy3456* |
|  | 53,163,164 | 47 | 13.9 | 45 | 55.6/17.3 | 55.5/5.9 | TAIWAN. Chiayi County: Mt. Yushan | 23°28'N, 120°55'E | 2,913 | *ccy3458* |
|  | 47,965,676 | 49 | 9.1 | 44 | 21.2/6.1 | 21.0/1.0 | TAIWAN. Kaohsiung City: Mt. Hsiaokuanshan | 23°09'N, 120°51'E | 2,446 | *ccy3338* |
| *C. kawakamii* | 57,811,986 | 47 | 17.1 | 45 | 68.6/31.0 | 68.4/6.5 | TAIWAN. Nantou County: Nenggao | 24°02'N, 121°15'E | 2,751 | *ccy3861* |
|  | 58,815,152 | 50 | 14.1 | 45 | 80.1/34.3 | 79.7/11.1 | TAIWAN. Nantou County: Mt. Xiaochilai | 24°08'N, 121°17'E | 3,040 | *ccy3544* |
| *C. pengii* | 57,811,986 | 46 | 26.7 | 45 | 91.1/38.7 | 90.9/17.0 | TAIWAN. New Taipei City: Mt. Lalashan | 24°43'N, 121°26'E | 1,736 | *ccy4054* |
|  | 53,346,610 | 45 | 22.4 | 44 | 90.2/45.9 | 89.7/21.1 | TAIWAN. New Taipei City: Mt. Lalashan | 24°43'N, 121°26'E | 1,736 | *ccy3995* |
| *C. suffultum* | 41,427,942 | 46 | 16.2 | 45 | 81.7/26.8 | 81.6/8.4 | JAPAN. Oita Prefecture: Yabakei Town | 33°29'N, 131°01'E | 1,142 | *ccy4209* |
| *C. nipponicum* var. *incomptum* | 52,207,200 | 46 | 19.1 | 44 | 84.2/42.6 | 83.8/10.8 | JAPAN. Fukuoka Prefecture: Ishigama | 33°29'N, 130°18'E | 250 | *ccy4207* |
| *C. kujuense* | 54,657,722 | 45 | 22.7 | 44 | 90.0/50.3 | 89.8/14.5 | JAPAN. Oita Prefecture: Minamitateishi | 33°17'N, 131°25'E | 1,250 | *ccy4214* |
| *Silybum marianum* | 32,523,260 | 44 | 29.7 | 44 | 85.8/45.7 | 85.4/21.2 | NCBI GenBank | -- | -- | SRR12539244 |
| *Cynara cardunculus* | 19,148,552 | 46 | 18.1 | 45 | 85.9/32.5 | 85.6/11.3 | NCBI GenBank | -- | -- | SRR16295441 |
| *Gerbera delavayi* | 28,597,092 | 45 | 21.8 | 44 | 91.9/50.1 | 91.9/24.1 | NCBI GenBank | -- | -- | SRR5480948 |
| *Nastanthus ventosus* | 26,354,302 | 46 | 21.5 | 45 | 83.1/44.1 | 82.7/20.3 | NCBI GenBank | -- | -- | SRR12034794 |

C: Complete BUSCOs; D: Complete and duplicated BUSCOs

Supp. table 2. Voucher material for pollen morphology.

| Taxa | **Location** | **Coordinate** | **Altitude** | **Date** | **Voucher** |
| --- | --- | --- | --- | --- | --- |
| *C. pengii* | TAIWAN. New Taipei City: Mt. Lalashan | 24°43'20.9"N, 121°26'31.2"E | 1,736 m | 14 Oct. 2023 | *C. Y. Chang 4308*, *4310* (TCF) |
| *C. kawakamii* | TAIWAN. Nantou County: Nenggao | 24°02'35.2"N, 121°15'60.0"E | 2,774 m | 27 Sept. 2022 | *C. Y. Chang 3863* (TCF) |
| *C. tatakaense* | TAIWAN. Nantou County: Zhongyang mine cabin | 23°29'11.8"N, 121°01'38.9"E | 2,849 m | 4 Sept. 2020 | *C. Y. Chang 3076* (TNM) |
|  | TAIWAN. Kaohsiung City: Mt. Hsiaokuanshan | 23°09'28.3"N, 120°51'42.7"E | 2,446 m | 20 Mar. 2021 | *C. Y. Chang 3338* (TNM) |

Supp. table 3. Voucher material for chromosome number analysis.

| Taxa | **Location** | **Coordinate** | **Altitude** | **Voucher** |
| --- | --- | --- | --- | --- |
| *C. pengii* | TAIWAN. New Taipei City: Mt. Lalashan | 24°43'20.9"N, 121°26'31.2"E | 1,736 m | *C. Y. Chang 3995*, *4344, 4346* (TCF) |
| *C. kawakamii* | TAIWAN. Nantou County: Mt. Xiaoqilai | 24°08'05.1"N, 121°17'25.4"E | 3,040 m | *C. Y. Chang 4343* (TCF) |

Supp. table 4. Comparison between Taiwanese and Japanese taxa of *Cirsium* subsect. *Nipponocirsium*.

| **Taxon** | *C. pengii* | *C. kawakamii* | *C. tatakaense* | *C. suffultum* | *C. nipponicum* var. *incomptum* | *C. kujuense* |
| --- | --- | --- | --- | --- | --- | --- |
| **Habit** | hermaphrodite | hermaphrodite | hermaphrodite | gynodioecious | hermaphrodite | hermaphrodite |
| **Leaf** | pinnatipartite | pinnatisect | pinnatisect | pinnatisect | pinnatisect | pinnatisect |
| **Capitula** | pot-shaped | bowl-shaped | bowl-shaped | bowl-shaped | bowl-shaped | pot-shaped |
| **Phyllary** | non-glutinous | non-glutinous | non-glutinous | non-glutinous | non-glutinous | glutinous |
| **Corolla** | bluish-purple | white | bluish-purple | bluish-purple | pale purple | bluish-purple |
| **Distribution** | endemic to Taiwan | endemic to Taiwan | endemic to Taiwan | endemic to Japan | endemic to Japan | endemic to Japan |
| **Reference** | this study | Chang et al. (2019) | Chang et al. (2019) | Kadota (2006) | Kadota (1995) | Kadota (2008) |
